# Which aspects facilitate the adherence of patients with low back pain to physiotherapy? A Delphi study

**DOI:** 10.1186/s12891-023-06724-z

**Published:** 2023-07-27

**Authors:** Andreas Alt, Hannu Luomajoki, Kerstin Luedtke

**Affiliations:** 1 Institute of Health Sciences, Department of Physiotherapy, Universität zu Lübeck, Lübeck, Germany; 2 Institute of physiotherapy, Zürich University of applied Sciences (ZHAW), Katharina-Sulzer-Platz 9, Winterthur, CH-8401 Switzerland

**Keywords:** Low back pain, Physiotherapy, Adherence, Expert consensus

## Abstract

**Background:**

The effectiveness of physiotherapy to reduce low back pain depends on patient adherence to treatment. Facilitators and barriers to patient adherence are multifactorial and include patient and therapist-related factors. This Delphi study aimed to identify an expert consensus on aspects facilitating the adherence of patients with back pain to physiotherapy.

**Method:**

International experts were invited to participate in a three-round standard Delphi survey. The survey contained 49 items (32 original and 17 suggested by experts) which were rated on 5-point Likert scales. The items were assigned to six domains. The consensus level was defined as 60%.

**Results:**

Of 38 invited experts, 15 followed the invitation and completed all three rounds. A positive consensus was reached on 62% of the 49 proposed items to facilitate adherence. The highest consensus was achieved in the domains “Influence of biopsychosocial factors” (89%) and “Influence of cooperation between physiotherapists and patients” (79%). Additional important domains were the “Influence of competencies of physiotherapists” (71%) and “Interdisciplinary congruence” (78%). “Administration aspects” and the “Use of digital tools” did not reach expert consensus.

**Conclusions:**

Biopsychosocial factors, therapeutic skills, and patient-physiotherapist collaboration should be considered in physiotherapy practice to facilitate adherence in patients with LBP. Future studies should prospectively evaluate the effectiveness of individual or combined identified aspects for their influence on patient adherence in longitudinal study designs.

## Background

According to national and international clinical guidelines, a patient with low back pain (LBP) attending physiotherapy is advised to perform regular physical exercises, avoid prolonged periods of rest, and long-term passive therapy measures such as manual therapy (MT) or massage [1, 34]. The long-term effects of LBP treatment depend on a complex process addressing cognition, function, and pain [10, 11, 14]. This can be achieved by physiotherapy approaches that facilitate patient self-management and require a high level of adherence [10, 11]. Adherence is defined as “the extent to which a person conforms to the agreed-upon recommendations of a health care provider” [30]. The term “adherence” emphasizes the concordant behavior of patient and physician [7] and thereby exceeds compliance, usually defined as “doing what the doctor said” [12]. In physiotherapy, the concept of adherence is multidimensional and based on biopsychosocial influences [2, 16, 18].

Previous research indicates that adherence, often referred to quantitatively as the level of adherence, can be influenced by several factors. These can concern the patient with LBP and be based on his level of motivation, self-discipline, acceptance of specific exercises, perceived effectiveness of the exercises, beliefs, and attitudes, cultural background, and communicative aspects [6, 8, 20–22, 24, 25]. Other factors are more related to the physiotherapist and include communication skills, motivation to enhance the self-efficacy of patients, building a physiotherapist-patient relationship, and professional experience [4, 13, 19, 21].

In a previously conducted focus group study, investigating the perspectives of patients and of physiotherapists, aspects influencing the adherence of patients with LBP were shown to be more complex than expected [3]. Patients requested long-term rehabilitation management, individualized therapy, and effective home programs to achieve a higher level of adherence. Physiotherapists requested more time for patient education. They indicated that adherence to physiotherapy in patients with LBP can be negatively influenced by the advice or expectations induced by other healthcare professionals. Physiotherapists and patients agreed that communication, the quality of the therapist-patient relationship, and individualized physiotherapy are essential factors facilitating adherence [3]. Following these personal insights into a selection of patients` and therapists` thoughts about adherence, this Delphi study aimed to identify a consensus of experts on adherence-facilitating aspects. The results of the Delphi study are intended to improve the understanding of how to facilitate adherence in patients with LBP to subsequently develop and evaluate targeted treatment strategies.

## Methods

A Delphi survey is a consensus method that solicits expert opinion through multiple rounds of questioning. It is characterized by different features: Anonymity, iteration, controlled feedback, and group response [28].

Among the various Delphi methods, the standard Delphi method was used in this study, including three rounds of questionnaires [28]. Data were collected from February 22 to April 01, 2023.

### Selection of delphi experts

The technique of purposive sampling was used to select informed individuals to serve on a panel of experts for the Delphi process [23, 31]. The experts were identified through a previously conducted systematic review aiming to identify tools to measure and evaluate the effectiveness of strategies to facilitate adherence in patients with LBP [2]. In addition to inviting the authors of publications included in this review, flyers were posted in physiotherapy groups on social media inviting physiotherapists to the study.

The competence of the experts to contribute to the consensus was based on predefined criteria (Table 1). To include the clinical and the research perspective on adherence, clinicians and researchers were invited to participate.

**Table 1:** Eligibility criteria

Inclusion criteria	Exclusion criteria
Researchers who have addressed adherence of patients with LBP in scientific articles **OR** At least 3 years of clinical experience in physiotherapy treatment of patients with LBP	Researchers exclusively investigating patient adherence to medication
	Researchers focusing on patients with psychological disorders
**AND** Ability to understand English (in writing)	Physiotherapists mainly treating patients with LBP in psychiatric settings

LBP = low back pain

All identified experts were contacted by e-mail and informed about study procedures and objectives. Those who expressed interest were given an informed consent form to read, sign, and return via e-mail.

### Instrument

**Fig. 1:**
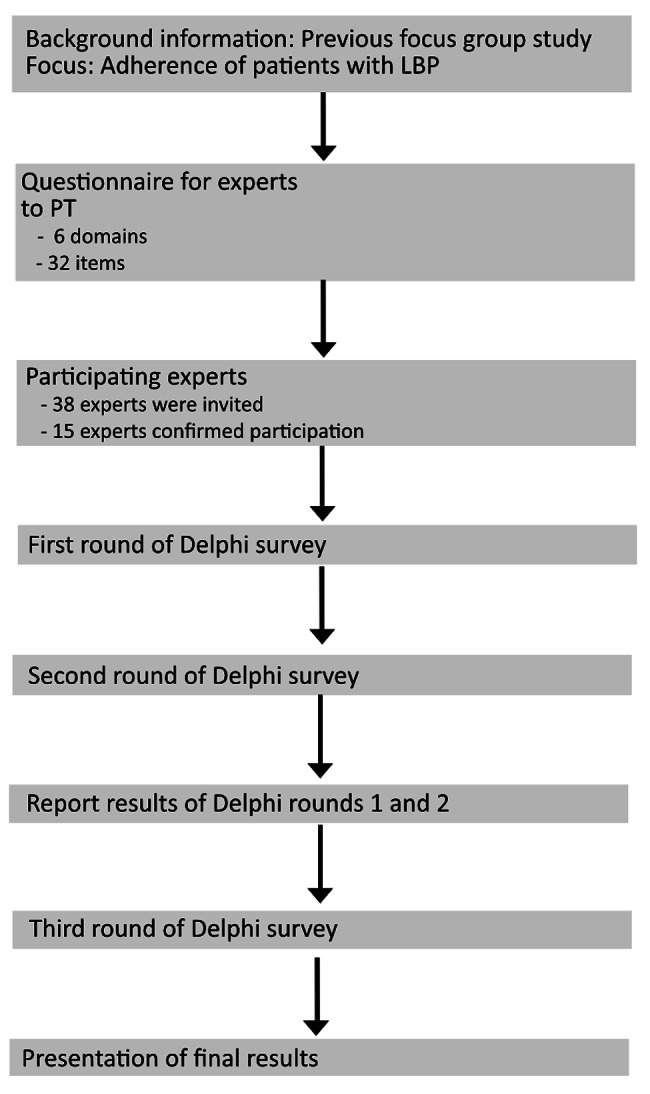
Methodology This figure shows the methodological structure of the Delphi study. It includes the preparation and the individual methodological steps

The first round of the Delphi survey consisted of three steps. First, participants were informed by e-mail how to complete the survey and how to rate the items. Then, participants received a questionnaire asking about their sociodemographic characteristics (Fig. 1). Finally, experts received the questionnaire with the domains and items related to the adherence of patients with LBP.

The questionnaire was developed based on a previously conducted systematic review [1] and items identified by patients and physiotherapists in a previously conducted focus group study [3]. The questionnaire for the first Delphi round consisted of six domains and 32 associated items potentially influencing adherence to physiotherapy, such as the influence of the biopsychosocial approach, the influence of cooperation between physiotherapists and patients, the influence of digitalization on adherence in patients with LBP (Table 2).

**Table 2:** Overview of the structure of the Delphi survey related to the first round

Domains		No. of items
1	The influence of the biopsychosocial approach on adherence of patients with LBP to PT	5
2	The influence of cooperation between physiotherapists and patients with LBP on their adherence to PT	6
3	Interdisciplinary congruence in therapeutic strategies influences the adherence of patients with LBP to PT	4
4	The influence of administrative aspects on the adherence of patients with LBP to PT	5
5	The influence of digitization on the adherence of patients with LBP to PT	6
6	The influence of competencies of physiotherapists on adherence of patients with LBP to PT	6
**Total number of items 32**		

LBP = low back pain; PT = physiotherapy

Experts rated the items of each domain on a 5-point Likert scale as absolutely correct [1], correct [2], don’t know [3], rather no [4], or wrong [5].

### Setting the consensus level

The Delphi method is based on selected participants reaching a consensus on a topic through multiple rounds of discussion. However, the opinions of experts can differ and 100% agreement on all issues is difficult to achieve. There is no recommendation on an appropriate level of agreement and different levels were chosen by previous authors [17, 28]. For this study, an item was excluded from subsequent rounds if more than 60% of the experts rated it as “rather no” or “wrong” (negative consensus). An item was included if 60% or more of the experts rated it as “absolutely correct” or “correct” (positive consensus). Items not reaching this level of agreement due to “don’t know” ratings, were presented as “no consensus”.

### Procedure for the delphi survey

**Fig. 2:**
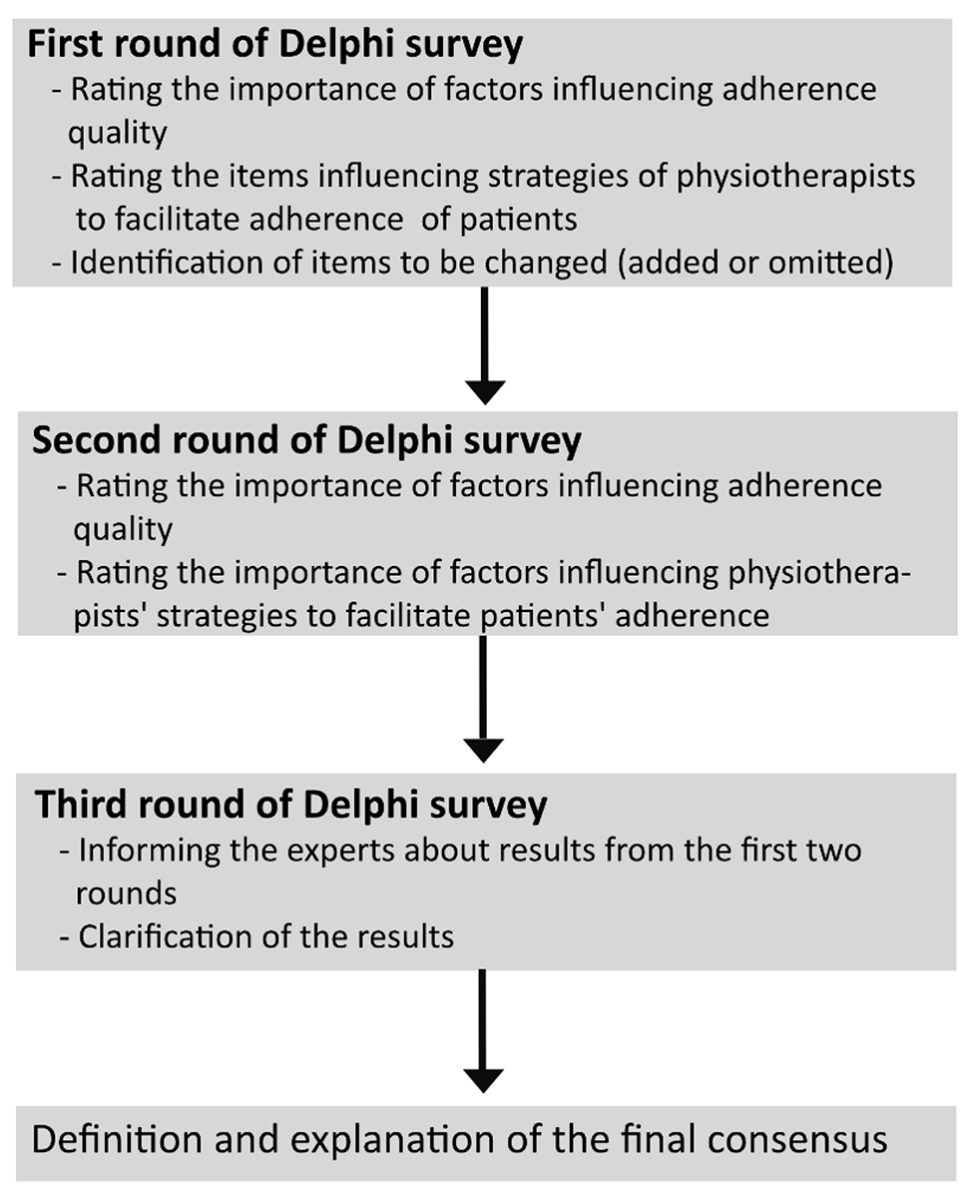
Delphi process This figure shows the contents of the individual Delphi rounds and their sequence

The Delphi survey included three rounds of questionnaires (Fig. 2). In the first round, participants were asked to rate the importance of items that influence the level of adherence of patients with LBP to physiotherapy. They could also name other items which they considered important.

The new items suggested by the experts in round one were included for expert ratings in the second round. In the second round, the experts rated the 17 new items which were also assigned to the six domains.

In the third round, all 15 participants were informed about the results from the first two rounds and asked to review whether they agreed with the results.

### Data analysis

The responses from each Delphi round were entered into a Microsoft Excel spreadsheet. To determine the consensus to include, the number of “absolutely correct” and “correct” ratings were counted and presented as a percentage of all ratings. In addition, open questions were asked in the first round for each dimension, which the experts could optionally answer. The answers of the experts to the open questions were converted into new items and presented to experts to be rated in the second round.

## Results

Out of 38 contacted experts, 15 agreed to participate in the Delphi survey. The experts were contacted via e-mail. 18 of the experts did not respond and five indicated they did not feel eligible. Participating experts came from six different countries, three continents, seven universities, eight physiotherapy centers, and had various professional positions (Table 3). The response rate in rounds one and two was 100% (n = 15). A positive consensus was reached on 62% of the 49 proposed items.

**Table 3:** Characteristics of experts

ID	Gender	Age (years)	AD	Country	Position	Specialization	PE (years)	Clinic. exp. with LBP	Scien. exp. with LBP	Prof. courses
E1	m	56	B.Sc.	GER	Employee	M.Sc. of NS	37	Yes	No	MI
E2	f	39	PhD	FIN	Lecturer, development expert	Research, teaching	16	Yes	Yes	MI, VC, IS
E3	f	30	M.Sc.	GER	Research associate, employee	Research, clinical practice	8	Yes	Yes	X
E4	m	29	Dipl.	GER	Employee, lecturer	Teaching, clinical practice	9	Yes	No	MI, CFT
E5	m	25	B.Sc.	CH	Employee	Clinical practice	4	Yes	No	MI
E6	m	36	M.Sc.	CH	Head of master programs	Research, lecturer	12	Yes	Yes	MI, PCC
E7	m	38	Dipl.	GER	Employee	Clinical practice	17	Yes	No	CFT
E8	m	56	Dipl.	GER	Management	LS, teaching, clinical practice, research	31	Yes	Yes	MI
E9	m	25	B.Sc.	GER	Employee	Teaching, clinical practice	3	Yes	No	MI
E10	m	50	M.Sc.	NL	Employer	IS, clinical practice	25	Yes	No	EP, MI, SPT
E11	f	46	Ph.D.	ZMB	Lecturer	Research, teaching	12	Yes	Yes	VC, LS, GPTR
E12	f	53	Ph.D.	USA	Lecturer	Research, teaching	27	No	Yes	CPS, HP
E13	f	34	Ph.D.	GER	Research associate	Research, teaching	10	Yes	Yes	X
E14	f	26	B.Sc.	GER	Employee	Clinical practice	5	Yes	No	MI, PCC
E15	f	46	Ph.D.	CH	Researcher	Research	12	Yes	Yes	X

AD = academic degree; B.Sc. = bachelor of science; CFT = cognitive functional therapy; CH = Switzerland; clinic. exp. = clinical experience; CPC = clinician-Patient Communication; EP = explain pain; f = female; FIN = Finland; GER = Germany; HP = Health Psychology; ID = identification of participant (coded); GPTR = gynecologic physiotherapy rehabilitation; IS = implementation science; LBP = low back pain; MI = motivational interviewing, m = male; M.Sc. = master of science; NL = Netherlands; PCC = patient centered communication; NS = neuroscience; PE = professional experience; Ph.D. = doctoral degree; scien. exp. = scientific experience; SPT = sports physiotherapy; USA = United States of America; VC = validating communication; ZMB = Zambia;

### Expert consensus for all domains

#### Domain one

The influence of the biopsychosocial approach on adherence of patients with LBP to physiotherapy.

Most experts (n = 13) indicated that applying a biopsychosocial approach influences adherence of patients with LBP and only two rated “don’t know”. All items in this domain in round one reached a high consensus to include (97%). For round two, four new items were suggested by experts for this domain, which all reached consensuses to include (Table 4).

**Table 4:** Consensus for domain one ”The influence of the biopsychosocial approach on adherence of patients with LBP to physiotherapy”

**The influence of the biopsychosocial approach on the adherence of patients with LBP to physiotherapy**											
**Ratings of experts round 1**											
Item		Absolutely correct		Correct		Don’t know	Rather no	Wrong	Included cons. (%)	Excluded cons. (%)	
1	**Acceptance of therapy program**	9		6					100		
2	**Explanation of therapy programs**	8		7					100		
3	Motivation of patients with LBP	12		2		1			93		
4	**Expectations of patients with**	13		2					100		
5	Beliefs of patients with LBP	13		1		1			93		
Positive consensus round 1 (mean)		97									
**Ratings of experts for newly suggested item in round 2**											
Item		Absolutely correct	Correct		Don’t know		Rather no	Wrong	Included cons. (%)	Excluded cons. (%)	
6	Understanding about a realistic course of treatment	12	1		1		1		87		
7	Health literacy of patients with LBP	8	6		1				93		
8	Safe surroundings in PT session	7	6		2				87		
9	ILC of patients	8	3		4				73		
10	Cultural situation of patients with LBP	1	9		4		1		67		
Number of experts (mean)		9	4		2		2				
Median of both rounds		8.5	4.5		1		1				
Positive consensus of round 2 (mean)		81									
Positive consensus both rounds (mean)		89									

LBP = low back pain; ILC = internal locus of control; PT = physiotherapy

#### Domain two

The influence of cooperation between physiotherapists and patients with LBP on their adherence to physiotherapy.

Most experts (n = 11) indicated with a consensus of 79% that the cooperation between physiotherapists and patients with LBP influences adherence. Three experts rated with “don’t know”. In round one, all items achieved a consensus to include except item “Opportunities of rating the PT quality”. Four new items were suggested by experts during round one and three of these were included according to the ratings from round two. Ratings for the item “Opportunities of rating the physiotherapy quality” had a high level of uncertainty (eight out of 15 experts rated “don’t know”) (Table 5).

**Table 5:** Consensus on domain two “The influence of cooperation between physiotherapists and patients with LBP on their adherence to physiotherapy”

**The influence of cooperation between physiotherapists and patients with LBP on their adherence to physiotherapy**														
**Ratings of experts round 1**														
Item		Absolutely correct			Correct		Don’t know	Rather no	Wrong			Included cons. (%)		Excluded cons. (%)
1	**Trust of patients with LBP**	15										100		
2	Patient-physio-therapist sympathy	5			7		3					80		
3	**Taking patients with LBP seriously**	14			1							100		
4	**Including the views of patients with LBP**	12			3							100		
5	Providing long-term updates	10			4		1					80		
6	**Verbal communication**	11			4							100		
Positive consensus round 1 (mean)		93												
**Ratings of experts for newly suggested items in round 2**														
Item			Absolutely correct	Correct		Don’t know		Rather no		Wrong	Included cons. (%)		Excluded cons. (%)	
1	Positively coined cues (verbal and non-verbal)		11	3		1					93			
2	Cultural factors influence adherence		1	10		4					73			
3	Understanding of morality by physiotherapists		3	11		1					93			
4	Opportunities of rating the PT quality		2	2		8		2		1			73	
Number of experts (mean)			8	5		3		2		1				
Median of both rounds			11	4		2								
Positive consensus round 2 (mean)			65											
Positive consensus both rounds (mean)			79											

LBP = low back pain; PT = physiotherapy

#### Domain three

Interdisciplinary congruence on therapeutic strategies influences adherence to physiotherapy of patients with LBP.

Most experts (n = 13) indicated that the influence of interdisciplinary congruence in terms of therapeutic strategies influences the adherence of patients with LBP. The highest consensuses to include in round one was achieved by the item “Therapeutic agreement” (100%). Two new items were suggested by experts during round one, both reaching consensus to include (Table 6).

**Table 6:** Consensus on domain three “Interdisciplinary congruence on therapeutic strategies influences adherence to physiotherapy of patients with LBP”

**Interdisciplinary congruence in therapeutic strategies influences adherence of patients with LBP to physiotherapy**														
**Ratings of experts round 1**														
Item		Absolutely correct			Correct		Don’t know	Rather no	Wrong			Included cons. (%)		Excluded cons. (%)
1	**Therapeutic agree-ment**	11			3			1				100		
2	Physician and therapist agreement	8			2		2	3				67		
3	**Regular professional exchange**	8			4		2	1				80		
4	Mutual professional respect	8			3		4					73		
Positive consensus round 1 (mean)		80												
**Ratings of experts for newly suggested items in round 2**														
Item			Absolutely correct	Correct		Don’t know		Rather no		Wrong	Included cons. (%)		Included cons. (%)	
1	Constant presence of respect towards colleagues		6	5		4					73			
2	**Similar evidence-based knowledge**		6	6		3					80			
Number of experts (mean)			9	4		3		2						
Median of both rounds			8	4		3		1						
Positive consensus round 2 (mean)			77											
Positive consensus both rounds (mean)			78											

LBP = low back pain

#### Domain four

The influence of administrative burdens on the adherence of patients with LBP to physiotherapy.

Responses for the five initial and two newly suggested items in this domain were controversial and consensus (to exclude) was reached for all items in the domain (Table 7).

**Table 7:** Consensus on domain four “The influence of administrative burdens on adherence of patients with LBP to physiotherapy”

**The influence of administrative burdens on the adherence of patients with LBP to physiotherapy**										
**Ratings of experts round 1**										
Item		Absolutely correct	Correct		Don’t know	Rather no	Wrong		Included cons. (%)	Excluded cons. (%)
1	The longer the wait for a PT appointment	2	2		5	5	1			73
2	Management of payers	3	3		5	4				60
3	Self-paying and adherence quality	1	1		3	5	5			87
4	Adherence to legally mandated timelines	2	4		6	3				60
5	Legally established procedures	3	2		5	3	2			67
Positive consensus round 1 (mean)										
**Ratings of experts for newly suggested items in round 2**										
Item		Absolutely correct		Correct	Don’t know	Rather no	Wrong	Included cons. (%)		Excluded. cons. (%)
6	Issuance of bills due to missed appointments affects adherence	1		3	6	5				73
7	Expensive PT influences adherence	4		4	6		1			47
Number of experts (mean)		2		3	5	4	2			
Median of both rounds		2		3	5	5	2			
Positive consensus round 2 (mean)										
Positive consensus both rounds (mean)										

LBP = low back pain; PT = physiotherapy

#### Domain five

The influence of digital tools in relation to physiotherapy on adherence of patients with LBP.

Ten experts stated that digital tools, e.g., the use of apps, influences the adherence of patients with LBP to physiotherapy. One expert did not rate items two and five. The consensus was reached that “Digital-based therapy (DBT) must be individualized” (93%) and for the use of graphs and trends. Two additional items were suggested in round one. These suggested that digital tools need to be manageable and that online recommendations can facilitate adherence. Both reached consensuses to include (Table 8).

**Table 8:** Consensus on domain five “The influence of digitization on adherence of patients with LBP”

The influence of digitization on adherence of patients with LBP to physiotherapy								
Ratings of experts round 1								
Item		Absolutely correct	Correct	Don’t know	Rather no	Wrong	Included cons. (%)	Excluded cons. (%)
1	Patients have no experience DBT		2	7	3	3		87
2	Privacy is not important to most patients		2	5	4	3		87
3	**DBT must be individualized**	8	6	1			93	
4	DBT variability promotes adherence	2	5	6	2			53
5	Graphs and trends improve adherence	6	6	1	1		80	
6	Adherence is higher to human-based PT than to DBT	4	4	4	2	1		47
Positive consensus round 1 (mean)		27						
**Ratings of experts for newly suggested items in round 2**								
Item		Absolutely correct	Correct	Don’t know	Rather no	Wrong	Included cons. (%)	Excluded cons. (%)
7	**The manageability of DBT improves adherence**	8	6	1			93	
8	Online recommendations improve adherence	3	9	2			80	
Number of experts (mean)		5	5	3	2	2		
Median of both rounds		5	6	3	2	3		
Positive consensus round 2 (mean)		87						
Positive consensus both rounds (mean)		57						

Cons = consensus; DBT = digital-based therapy; LBP = low back pain; PT = physiotherapy

#### Domain six

The influence of competencies of physiotherapists on adherence of patients with LBP.

Most of the experts (n = 13) stated that the competence of physiotherapists influences the adherence of patients with LBP to physiotherapy. One expert did not rate the item “Offering sufficient HP”. All six proposed items on physiotherapist-related aspects reached a consensus to include. The two new proposed items on the reputation of physiotherapists and regular supervision by other physiotherapists were not included in the consensus due to a high number of “don’t know” ratings (Table 9).

**Table 9:** Consensus on domain six “The influence of competencies of physiotherapists on adherence of patients with LBP”

**The influence of competencies of physiotherapists on adherence of patients with LBP to physiotherapy**														
**Ratings of experts round 1**														
Item		Absolutely correct			Correct		Don’t know	Rather no	Wrong			Included cons. (%)		Excluded cons. (%)
1	Motivation of physio-therapists	11			2		1	1				87		
2	**Good knowledge or courses**	12			3							100		
3	**Communication skills**	14			1							100		
4	**Individual patient-oriented PT strategy**	13			2							100		
5	Offering sufficient HP	7			6			1				87		
6	Authenticity of physio-therapists	7			7		1					93		
Positive consensus round 1 (mean)		98												
**Ratings of experts for newly suggested items in round 2**														
Item			Absolutely correct	Correct		Don’t know		Rather no		Wrong	Included. cons. (%)		Excluded cons. (%)	
7	Regular supervision of physiotherapists by other physiotherapists		5	3		5		2					47	
8	Reputation of therapist		2	2		8		2		1			73	
Number of experts (mean)			9	4		4		2		1				
Median of all rounds			9	3		3		2						
Positive consensus of round 2 (mean)			71											
Positive consensus in both rounds (mean)			71											

Cons. = consensus; LBP = low back pain; pos. = positive; PT = physiotherapy

In the third and final round, the experts were informed about the results from the first two rounds. They were asked whether they agreed with the summary of responses and to comment on the results. No adjustments were required from round three.

## Discussion

The purpose of this Delphi study was to reach an expert consensus on aspects to include when aiming to facilitate adherence to physiotherapy in patients with low back pain. Six domains were developed containing six to ten items (total of 49 items) of which 17 were contributed by experts during round one. The highest consensus (100%) was reached for items within the domains one, two, three, and six. This indicated that the influence of interprofessional collaboration (four items at 100% consensus), a biopsychosocial approach, and the competencies of physiotherapists (three items at 100% consensus each), as well as the patient-therapist relationship, were regarded as the most relevant factors influencing patient adherence.

The high consensus reached for all items describing a positive patient-therapist relationship, is in line with findings from qualitative studies. These reported that the relationship between the patient and the healthcare provider, e.g., the physiotherapist is of high importance [3, 6, 22, 26]. Participation, commitment, negotiation, and sometimes compromise improve the responsibility of the patient and thus the basis for adherence [22].

The relevance of interdisciplinary congruence, mentioned in domain three, was also identified in our previously conducted focus group study. Physiotherapists argued that the advice and information provided by other healthcare providers, influenced the expectations of patients and thereby their adherence (positively or negatively) [3].

Indications for the importance of this aspect have been reported in other qualitative studies [19, 21]. Studies using quantitative approaches postulated the use of communication strategies, individualized patient-centered physiotherapy, and knowledge of the evidence for treatment options [9, 20]. Communication as a method to influence adherence was also researched in the RCT by Londsdale et al. (2017) [20]. They found that communication skills of physiotherapists had short-term positive effects on self-reported home-based adherence of patients (weeks 1–12) but not on other adherence factors, e.g., adherence to back exercises. Coppack et al. (2012) showed in their RCT that the level of adherence in the group with goal-setting (group 1) was significantly higher than in the two comparison groups (group 2 = standard exercise program with motivation; group 3 = standard exercise program with monitoring of exercise technique for safety) [9]. But they did not present information about the specific reason for the superior results of the group with goal-setting.

Less information was available for aspects related to “digitalization” [29, 32], “administrative burdens” [15], and their influence on adherence. This could explain the relatively high number of “don’t know” ratings. Simple methods of DBT, such as the use of video games that promote activity, have been shown by the existing literature to effectively influence adherence in patients with LBP [29, 31]. In this current sample of experts, there was agreement that digital tools need to be individualized [5, 26], easy to manage, and should provide graphics and trends to increase motivation. Online recommendations were also regarded to facilitate adherence. Zhang et al. (2019) reported that media campaigns can influence patient health information seeking and that health information seeking can influence patient adherence [33]. There is currently no additional evidence for a relationship between adherence and online health information.

Whether administration aspects influence adherence was perceived controversially. While a burden to patients and therapists it may not have an influence on adherence to physiotherapy. Herd et al. (2021) noted that administrative burden depends on many factors, such as access to healthcare, appointment management, and costs. For patients with chronic conditions, these factors might accumulate to a burden influencing adherence to physiotherapy. In contrast to the findings from our focus group study, the experts did not recognize self-paying of patients with LBP for physiotherapy as an aspect influencing adherence [3].

This Delphi study provides expert consensus on aspects that facilitate the adherence of patients with LBP to physiotherapy. Future research has to evaluate in prospective longitudinal study designs whether individual aspects or combinations of these are the most effective to facilitate adherence to physiotherapy.

### Limitations

The suggestions emerging from this Delphi survey are based on a small number of experts. The experts came from six different countries and three continents (North America, Africa, and Europe). However, they do not represent the general population of physiotherapists. The study cannot provide evidence for the effectiveness of one or more of the proposed strategies.

## Conclusion

Biopsychosocial aspects, implemented into physiotherapy treatment, but also the competencies of physiotherapists, interprofessional congruence, and the patient-therapist relationship were seen as important aspects to influence adherence. The use of digital tools could facilitate adherence if designed to meet the individual needs of patients. Whether administrative aspects influence adherence is unclear. Longitudinal studies evaluating the effect of using the identified items are required to assess whether patient adherence can be influenced using these strategies and which strategy results in the best outcomes.

## Data Availability

All data generated or analyzed during this study are included in this published article.
